# Effectiveness of indoor residual spraying on malaria control: a systematic review and meta-analysis

**DOI:** 10.1186/s40249-022-01005-8

**Published:** 2022-07-23

**Authors:** Yiguo Zhou, Wan-Xue Zhang, Elijah Tembo, Ming-Zhu Xie, Shan-Shan Zhang, Xin-Rui Wang, Ting-Ting Wei, Xin Feng, Yi-Lin Zhang, Juan Du, Ya-Qiong Liu, Xuan Zhang, Fuqiang Cui, Qing-Bin Lu

**Affiliations:** 1 Department of Laboratorial Science and Technology & Vaccine Research Center, School of Public Health, Peking University, No. 38, Xueyuan Road, Haidian District, Beijing, 100191 People’s Republic of China; 2 Department of Public Health Emergency Management, School of Public Health, Peking University, Beijing, 100191 People’s Republic of China; 3 Institute for Hospital Management Research, Chinese PLA General Hospital, Beijing, 100039 People’s Republic of China; 4 Global Center for Infectious Disease and Policy Research & Global Health and Infectious Diseases Group, Peking University, Beijing, 100191 People’s Republic of China

**Keywords:** Indoor residual spraying, Malaria, Meta-analysis, Effectiveness evaluation

## Abstract

**Background:**

Indoor residual spraying (IRS) is one of the key interventions recommended by World Health Organization in preventing malaria infection. We aimed to conduct a systematic review and meta-analysis of global studies about the impact of IRS on malaria control.

**Method:**

We searched PubMed, Web of Science, Embase, and Scopus for relevant studies published from database establishment to 31 December 2021. Random-effects models were used to perform meta-analysis and subgroup analysis to pool the odds ratio (*OR*) and 95% confidence interval (*CI*). Meta-regression was used to investigate potential factors of heterogeneity across studies.

**Results:**

Thirty-eight articles including 81 reports and 1,174,970 individuals were included in the meta-analysis. IRS was associated with lower rates of malaria infection (*OR* = 0.35, 95% *CI*: 0.27–0.44). The significantly higher effectiveness was observed in IRS coverage ≥ 80% than in IRS coverage < 80%. Pyrethroids was identified to show the greatest performance in malaria control. In addition, higher effectiveness was associated with a lower gross domestic product as well as a higher coverage of IRS and bed net utilization.

**Conclusions:**

IRS could induce a positive effect on malaria infection globally. The high IRS coverage and the use of pyrethroids are key measures to reduce malaria infection. More efforts should focus on increasing IRS coverage, developing more effective new insecticides against malaria, and using multiple interventions comprehensively to achieve malaria control goals.

**Supplementary Information:**

The online version contains supplementary material available at 10.1186/s40249-022-01005-8.

## Background

Malaria is an insect-borne disease caused by *Plasmodium* parasite infection through the bite of infected mosquitoes, which was endemic in 87 countries and contributed to approximately 241 million cases and more than 627,000 deaths globally in 2020 [1]. Although the epidemiological burden of malaria has decreased significantly during 2000–2019 for the annual incidence (from 81/1000 to 56/1000 population at risk) and mortality (from 30/100,000 to 13/100,000 population at risk), it remains a major public health concern globally, especially in Africa, where the deaths caused by malaria accounted for about 95% of deaths globally [1].

In the past decades, numerous measures have been developed and implemented to prevent the malaria epidemic. Between 2000 and 2015, at least 663 million malaria cases were estimated to be averted by using malaria control interventions, vector control measures in particular [2]. Indoor residual spraying (IRS) is a key component in vector control of malaria, which has been used and showed the effectiveness in a variety of countries [3]. IRS works via spraying a long-lasting residual insecticide to internal and exterior surfaces of a house where malaria vectors might rest and be killed by the insecticide [4]. In the 1930s, IRS with pyrethrum succeed on malaria control in South Africa and India [5]. Between the 1940s and the 1960s, several pilot projects performed in African countries aimed at eliminating malaria demonstrated that malaria could be highly responsive to control by IRS with insecticides. In addition, the goal of eliminating malaria has been achieved in the United States and some European countries by using IRS insecticides such as dichloro-diphenyl-trichloroethane (DDT) [6]. On 30 June 2021, China was certified by the World Health Organization (WHO) as a malaria-free country with 4 consecutive years of reporting no indigenous cases [7].

In recent years, most studies in African countries indicated that IRS was associated with reductions in the incidence of malaria [8–12]. For example, after three rounds of IRS with bendiocarb from December 2014 to December 2015 in Tororo, Uganda, the significantly lower incidence of malaria and prevalence of parasitemia were observed in the following investigations [8]. Another study in Uganda also showed the same association between IRS implementation and a lower incidence of malaria, though a waned reduction effect in malaria occurred 4 months following IRS [9]. However, the effectiveness of IRS was not consistent across studies. A study carried out in northern Zambia reported that IRS with pirimiphos-methyl contributed to 25% of decline in parasite prevalence during rainy seasons, while no such decline existed in dry seasons [13].

Although IRS might be a useful measure to control malaria, its coverage remains extremely low in malaria-endemic countries. According to the WHO report, the percentage of the population susceptible to malaria protected by IRS at the globe declined from 5.8% in 2010 to 2.6% in 2020 [1]. Low IRS coverage might have unfavorable effects on the progress towards global eradication of malaria. Thus, we need to pool existing evidence on the effectiveness of IRS to prevent malaria so as to inform intervention decisions and practices in malaria control. A previous systematic review and meta-analysis published in 2012 included 13 studies and indicated a summary risk reduction of 62% for malaria following the implementation of IRS [14]. In light of the limited number of original studies pooled and the lack of subgroup analysis in the previous meta-analysis, it is imperative to perform an updated one to provide more robust and comprehensive information by incorporating over 20 recent extra published literature and carrying out more in-depth and detailed analysis. In this study, we aimed to estimate the effect of IRS on malaria control based on all the related studies and analyze potential impact factors of IRS’s effectiveness.

## Methods

This systematic review and meta-analysis were conducted following the principles of Preferred Reporting Items for Systematic Reviews and Meta-Analyses (PRISMA) [15].

### Literature retrieval and selection criteria

We searched systematically for relevant studies published from database establishment to 31 December 2021 from PubMed, Web of Science, Embase, and Scopus. The searching strategy consisted of a combination of keyword items in titles or abstracts as follows: Malaria AND (Indoor residual spraying OR IRS OR Indoor residual spray) AND (effectiveness OR protection OR prevalence OR incidence OR rate OR ratio OR proportion). These keywords relevant to the study aims were determined according to the discussion among the authors and the retrieval strategies used in previous systematic reviews on malaria and epidemiological outcomes [14, 16]. In addition, reference lists of original studies included were checked for potential missed studies in database searches. We did not contact any authors for providing additional original data.

All studies obtained through the initial search were entered into EndNote version X9 (Clarivate, Philadelphia, Pennsylvania) to remove duplicates automatically. Two researchers YZ and MX independently carried out the screening of titles and abstracts, followed by a full-text check for remaining papers. Discrepancy in screening results was resolved by discussion in the two researchers and a consultation with another experienced researcher. Studies were selected for data extraction and subsequent data analysis if they met criteria concurrently as follows: (1) malaria was the target disease; (2) IRS was the only intervention measure; (3) authors reported the detailed number of cases and number of total population in the intervention group and the control group, or these values could be recalculated based on existing data in results; (4) the impact of IRS on malaria was assessed through before-after self-control or setting up another control group without IRS implementation; (5) published in English. Eligibility of original studies was also assessed in accordance with several exclusion criteria as follows: (1) being a review, conference abstract, comment, or case report; (2) only reporting outcomes of entomological indicators; (3) reporting results from mathematical modelling other than data in the real world; (4) without estimating the impact of IRS on malaria or related indicators. In addition, when multiple studies reported results from the same resource population, studies with smaller sample sizes or shorter follow-up periods were excluded.

### Quality assessment and data extraction

Quality assessment of original studies successfully passing the full-text screening was done using the Joanna Briggs Institute (JBI) Critical Appraisal tools checklist for analytical cross-sectional studies and checklist for quasi-experimental studies [17]. The two appraisal tools respectively included 9 and 10 items associated with study design and quality control. Studies with more than 50% of items met were regarded as eligible for further data analysis [18]. YZ and WZ independently carried out the quality assessment, and disagreement was addressed through discussion.

Data were extracted independently by YZ and MX with a predefined and standardized form, including study variables when available as follows: first author, publication year, study design, type of control (a before-after self-control or a blank control), study location, study population, malaria epidemic level, outcome indicator, malaria diagnosis method, type of IRS insecticide, frequency of IRS, IRS coverage, coverage of bed net, time of IRS implementation, time of IRS effectiveness evaluation, effect size [odds ratio (*OR*), risk ratio (*RR*), incidence rate ratio (*IRR*), and rate difference (*RD*)] indicating IRS impact and its 95% confidence interval (*CI*), and the number of cases and the number of total population in both intervention group and control group. Multiple records were extracted when there were multiple reports of targeted outcomes involving different investigation time points and locations. In addition, we accessed and documented the gross domestic product (GDP) in 2019 from Trading Economics website [19] and malaria incidence rate in 2019 from the website of WHO of the countries involved in original studies in this review to perform subgroup analyses.

### Statistical analysis

The pooled *OR* and RR with 95% *CI* were used to evaluate the association between IRS and malaria risk. Cochran’s Q and *I*^2^ statistics were used to estimate the heterogeneity among the studies [20]. *I*^2^ < 25% and *I*^2^ of 25–75% respectively denoted low heterogeneity and moderate heterogeneity, and *I*^2^ > 75% was regarded as high heterogeneity. A random-effects model with Mantel–Haenszel method was used to do all the meta-analyses in light of high heterogeneity appeared across studies. Results were visualized through mapping forest plots. Some variables were used for subgroup analysis in light of heterogeneity, including study design, GDP in corresponding country (< 30 billion USD, 30–60 billion USD and ≥ 60 billion USD), incidence rate per 1000 population at risk (< 250 per 1000 and ≥ 250 per 1000), malaria epidemic level, IRS coverage (< 80% and ≥ 80%), bed net coverage (0%, 0–50%, 50–90%, ≥ 90% and unknown), and IRS chemicals. Subgroup analysis was only performed on datasets containing at least two studies. Meta-regression model was performed to compare the effects of IRS on malaria among different study-level variables. Sensitivity analysis was performed to strengthen reliability of the result by carrying out meta-analyses omitting each study to examine whether there was a study with disproportionately excessive impact. In addition, only the cross-sectional/case-control studies and only the cohort/randomized controlled trial (RCT) studies were kept to respectively calculate a pooled *OR* and *RR* in order to evaluate the stability of results. Funnel plot and Egger's test were used to assess the potential bias of publication. *P* < 0.05 (two-sided) was defined as statistically significant. All data analyses were performed using Stata 17.0 (Stata Corp LP, College Station, TX, USA).

## Results

### Overview of the included studies

**Fig. 1:**
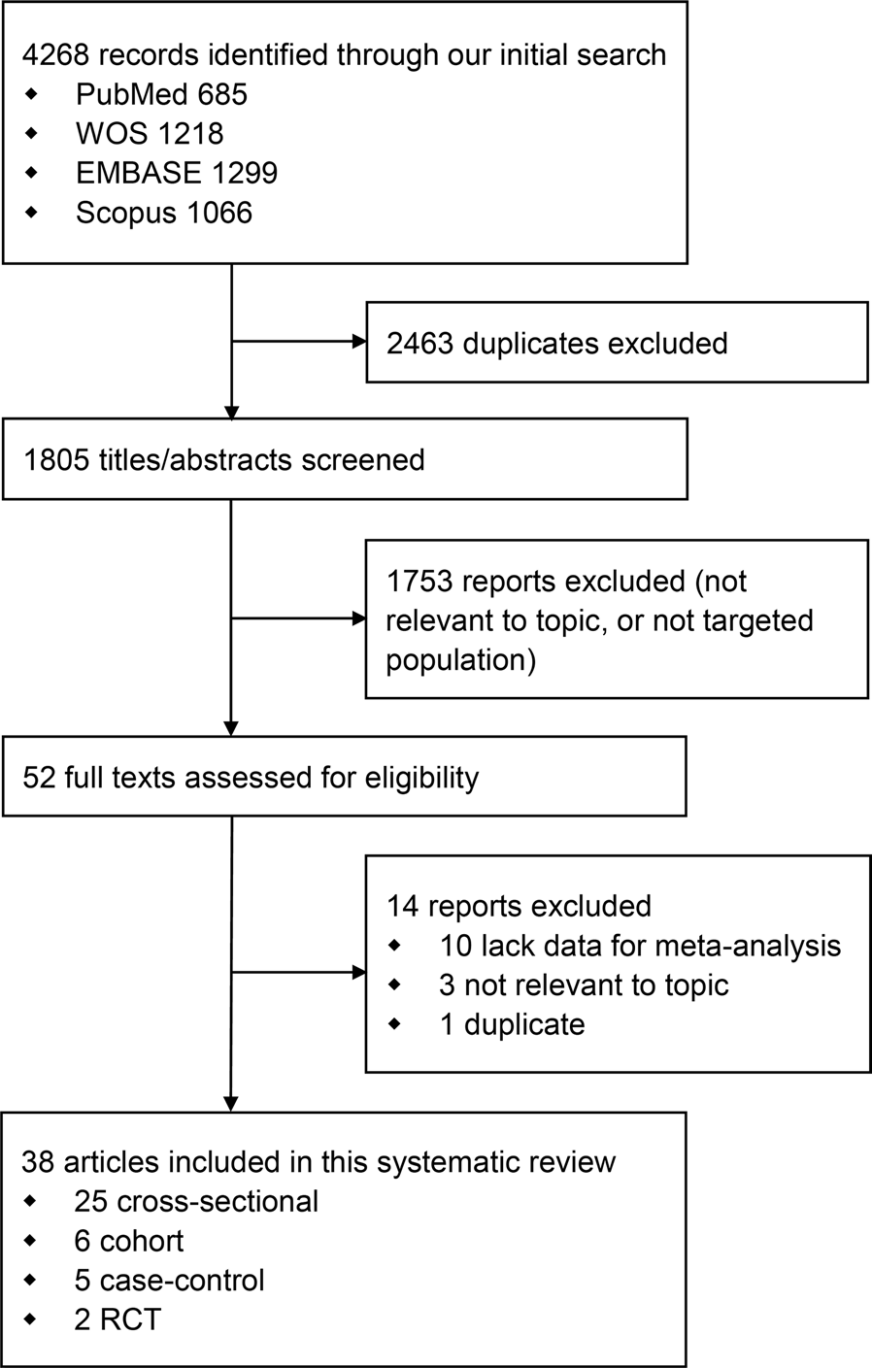
Selection of reports for inclusion in systematic review and meta-analysis. *WOS* Web of Science, *RCT* randomized controlled trial

Among the 4268 records initially searched in electronic databases, 2463 duplicates in EndNote software, 1753 reports in screening of titles and abstracts, and 14 reports in screening of full texts, were removed. A total of 38 articles (81 reports) were included in the final analysis, composed of 25 cross-sectional studies, six cohort studies, five case-control studies, and two RCT studies (Fig. 1).

**Fig. 2:**
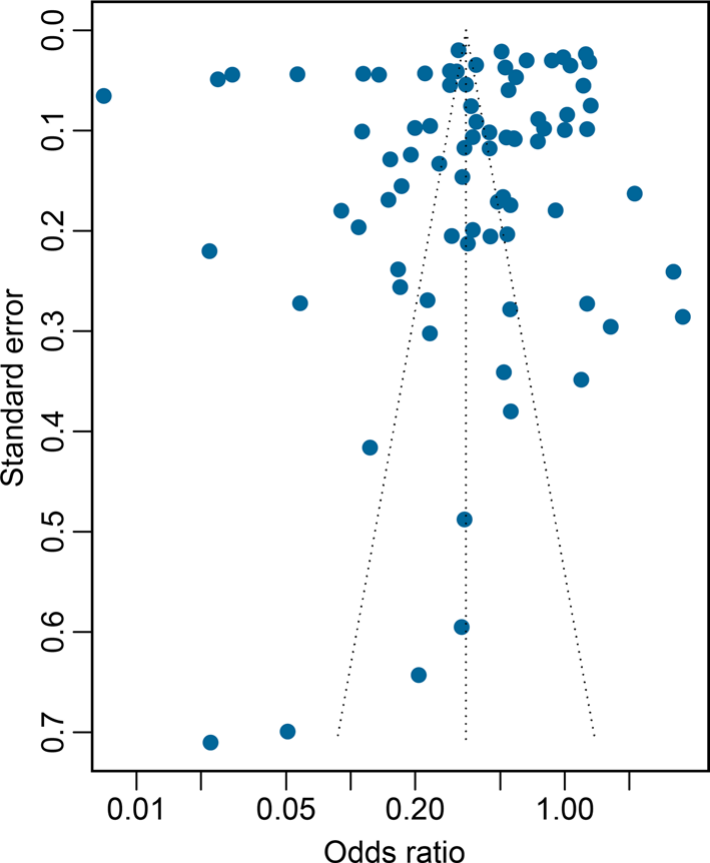
Funnel plot of 81 reports in the meta-analysis

Results of quality assessment showed 36 observational studies fulfilled at least 5 items (5/8, 62.5%) of all items and they were all included. Two RCT studies fulfilled at least 8 items (8/9, 88.9%) and were also included (Additional file 1: Tables S1 and S2). The funnel plot presented symmetrical distribution of all studies, and the Egger’s test did not show any statistical significance (*P* = 0.221). Therefore, a low risk of publication bias was observed across studies in this systematic review (Fig. 2).

Of the 38 original articles included, 35 were carried out in African countries and only three were in India (Table 1). Twenty-eight were published after the year of 2010, and 19 focused on children. Rapid diagnostic test (*n* = 23) was the most frequent method used to diagnose malaria, followed by blood smear test (*n* = 13) and clinical judgement (*n* = 2). Pyrethroids (*n* = 13) were the most common IRS insecticide used in articles, followed by the use of multiple insecticides (*n* = 12) and DDT (*n* = 4). In addition, 19 articles reported an IRS coverage at least 80%, 7 reported an IRS coverage less than 80%, and 12 did not report the value.

**Table 1:** Characteristics of original studies and the study populations

First author	Publication year	Study design	Study country	Study location	Population	Malaria epidemic level	Outcome	Diagnosis method	IRS drug	IRS coverage
Jambou	2001	Cross-sectional	Madagascar	168 municipalities	Children (mean 8.4 years)	High	Malaria parasites prevalence	Blood smear test	DDT	Unknown
Guyatt	2002	Cross-sectional	Kenya	Gucha District	General population	High	*Plasmodium falciparum* infection	RDT	Pyrethroids	Unknown
Gunasekaran	2005	Cross-sectional	India	Intervention: 54 villages Control: 10 villages	General population	High	*Plasmodium falciparum* infection	Blood smear test	DDT	≥ 80%
Sintasath	2005	Cross-sectional	Eritrea	2779 households (12,937 individuals) from 5 zobas	General residents (except children aged < 1 month)	Low	*Plasmodium falciparum* and *Plasmodium vivax* infection	RDT	Multiple	Unknown
Singh	2006	Cross-sectional	India	40 villages	Children ≤ 10 years; > 10 years	High	Malaria	RDT, blood smear test	Pyrethroids	≥ 80%
Kleinschmidt	2006	Cross-sectional	Equatorial Guinea	15 sentinel sites	2–15 years	High	*Plasmodium falciparum* infection	RDT, blood smear test (PCR)	Multiple	≥ 80%
Protopopoff	2008	Cross-sectional	Burundi	4 zones	1–9 years, > 9 years	High	Malaria infection, high-density parasitemia, clinical malaria	Blood smear test, RDT	Multiple	≥ 80%
Tseng	2008	Cohort	South Sudan	All districts	Children aged < 9 years	High	Malaria parasitemia	Blood smear test	Pyrethroids	≥ 80%
Bukirwa	2009	Cross-sectional	Uganda	Kanungu District	General population	Medium	Clinical malaria	Microscopy	Pyrethroids	≥ 80%
Zhou	2010	Cohort	Kenya	1100 houses	Children aged < 14 years	High	Malaria incidence, *Plasmodium* parasite infection, *Plasmodium* parasite prevalence	RDT, blood smear test	Pyrethroids	≥ 80%
Rehman	2011	Cross-sectional	Malawi	14 sentinel sites	< 15 years	High	Malaria	RDT, blood smear test	Pyrethroids	< 80%
Rehman	2011	Cross-sectional	Mainland Equatorial Guinea	2 provinces	< 15 years	High	Malaria	RDT, blood smear test	Multiple	< 80%
Aregawi	2011	Cross-sectional	Zanzibar	6 inpatients facilities out of 7 in Zanzibar	General population	High	Malaria	Clinical judgement	Pyrethroids	≥ 80%
Hamusse	2011	Cross-sectional	Ethiopia	22 sprayed and 22 unsprayed villages	General population	High	Malaria incidence	Blood smear test	DDT	≥ 80%
Skarbinski	2012	Cross-sectional	Malawi	1 district (Nkhotakota District)	Children aged < 5 years	High	Malaria parasitemia	Blood smear test	Pyrethroids	≥ 80%
Fullman	2013	Cross-sectional	17 countries in sub-Saharan Africa	NA	Children aged < 5 years	High	Parasitemia	RDT and/or blood smear test	Multiple	Unknown
Steinhardt	2013	Cross-sectional	Uganda	3 districts	0–59 months	High	Parasite prevalence	RDT	Multiple	≥ 80%
Mashauri	2013	Cross-sectional	Tanzania	6 villages	Children aged < 5 years	High	Malaria parasitemia	Blood smear test	Multiple	Unknown
Mashauri	2013	Cross-sectional	Tanzania	6 villages	Children aged 5–14 years	High	Malaria parasitemia	Blood smear test	Multiple	Unknown
Mashauri	2013	Cross-sectional	Tanzania	6 villages	Children aged ≥ 15 years	High	Malaria parasitemia	Blood smear test	Multiple	Unknown
West	2013	Cross-sectional	Tanzania	68 villages	Children aged 0.5–14 years	Medium	*Plasmodium falciparum* infection	RDT	Pyrethroids	≥ 80%
Gimnig	2016	Cross-sectional	Kenya	2 districts	General population	High	Clinical malaria	Parasitemia with fever	Pyrethroids	< 80%
Hamainza	2016	Cross-sectional	Zambia	165 households in districts of Luangwa and Nyimba	General population	High	Malaria	RDT	Multiple	< 80%
Kesteman	2016	Case-control	Madagascar	31 sentinel health centres	General population	High	Clinical malaria	RDT or microscopy	Pyrethroids	< 80%
Odugbemi	2016	Cross-sectional	Nigeria	20 local government areas	< 5 years	High	Parasitemia	RDT	Pyrethroids	≥ 80%
Kesteman	2016	Cross-sectional	Madagascar	4 southern study sites	Children aged 0.5–14 years	Low	*Plasmodium* infection	RDT	Pyrethroids	Unknown
Kesteman	2016	Cross-sectional	Madagascar	21 of all targeted zones except the south	Children aged 0.5–14 years	Low	*Plasmodium* infection	RDT	Pyrethroids	Unknown
Wanzira	2017	Cross-sectional	Uganda	210 areas	Children aged < 5 years	High	Malaria parasitemia	Blood smear test	Methyl carbamate	Unknown
Raouf	2017	Cross-sectional	Uganda	City (Apac District)	< 14 years	High	Malaria	Microscopy or RDT	Multiple	≥ 80%
Rek	2018	Cohort	Uganda	Subcounty	0.5–11 years	High	Parasite prevalence, malaria incidence	Blood smear test	Methyl carbamate	Unknown
Hast	2019	Cross-sectional	Zambia	Nchelenge District	General population	High	*Plasmodium falciparum*	RDT	Multiple	≥ 80%
Nankabirwa	2019	Cohort	Uganda	Subcounty	0.5–10 years and ≥ 18 years	High	Microscopic parasitemia	Blood smear test	Unknown	Unknown
Loha	2019	RCT	Ethiopia	44 villages	General residents	High	Malaria incidence, anemia	RDT	Methyl carbamate	≥ 80%
Tugume	2019	Cohort	Uganda	1 district	≥ 18 years	High	Malaria	RDT, blood smear test	Pirimiphos-methyl	≥ 80%
Arinaitwe	2020	Case-control	Uganda	1 hospital	General population with a history of recent overnight travel	Low	Malaria	RDT	Pirimiphos-methyl	Unknown
Habyarimana	2020	Cross-sectional	Rwanda	Village	Children aged 6 months to 14 years	High	Malaria	RDT	Pyrethroids	< 80%
Kamya	2020	Cohort	Uganda	Tororo District	Children aged 6 months to 2 years	High	Parasitemia	Microscopy, PCR	Multiple	≥ 80%
Wubishet	2021	Case-control	Ethiopia	1 district	General population	High	Malaria	RDT	Methyl carbamate	≥ 80%
Smith	2021	Case-control	The Republic of Nabimia	1 district (Zambezi River region)	Residents aged < 76 years	High	Parasite, *Plasmodium falciparum*	RDT	DDT	< 80%
Siegert	2021	Case-control	India	1 district (Mangaluru)	Residents aged > 18 years	Low	*Plasmodium* infection	PCR	Multiple	< 80%
Chaccour	2021	RCT	Mozambique	Rural Mopeia District	Children aged < 5 years	High	Malaria	RDT	Pirimiphos-methyl	Unknown
Fekadu	2021	Cross-sectional	Ethiopia	Health center	Patients in Heben Arsi District	Medium	Malaria	Blood smear test	Methyl carbamate	Unknown

*RCT* randomized controlled trial, *RDT* rapid diagnostic test, *PCR* polymerase chain reaction, *DDT* dichloro-diphenyl-tricgloroethane

### Overall effect of IRS on malaria prevention

**Fig. 3:**
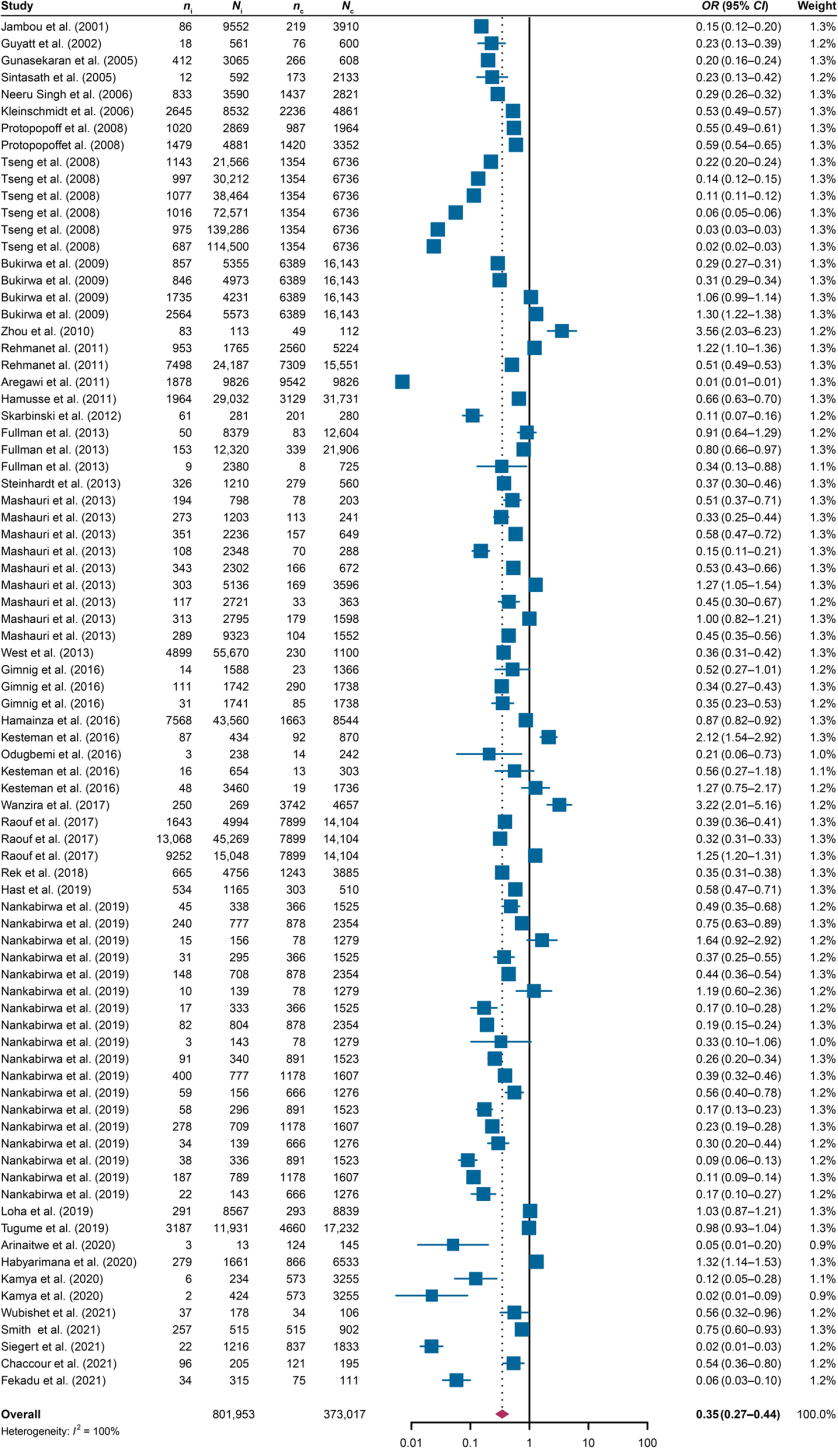
The total effect of indoor residual spraying on the risk of malaria by the random effects model. *n*_i_: the number of malaria cases who accepted indoor residual spraying (IRS); *N*_i_: the number of people who accepted IRS; *n*_c_: the number of malaria cases who did not accept IRS; *N*_c_: the number of people who did not accept IRS; *OR*: odds ratio; *CI*: confidence interval

This meta-analysis of 81 reports from 38 relevant articles [8, 10–13, 21–53] included a pooled study population that contained 1,174,970 individuals, with 801,953 individuals accepting IRS and 373,017 living without IRS. The combined *OR* based on a random-effects model for the association between IRS and the risk of malaria was estimated as 0.35 (95% *CI*: 0.27–0.44, *I*^2^ = 100%) (Fig. 3). Of the 81 reports, only 17 showed a crude odds ratio with upper limits of 95% *CI* passing one, denoting an unrelated or positive relationship between IRS and the risk of malaria. Most studies showed a protective effect for IRS on the risk of malaria.

### Subgroup meta-analysis on the effect of IRS on malaria prevention

**Fig. 4:**
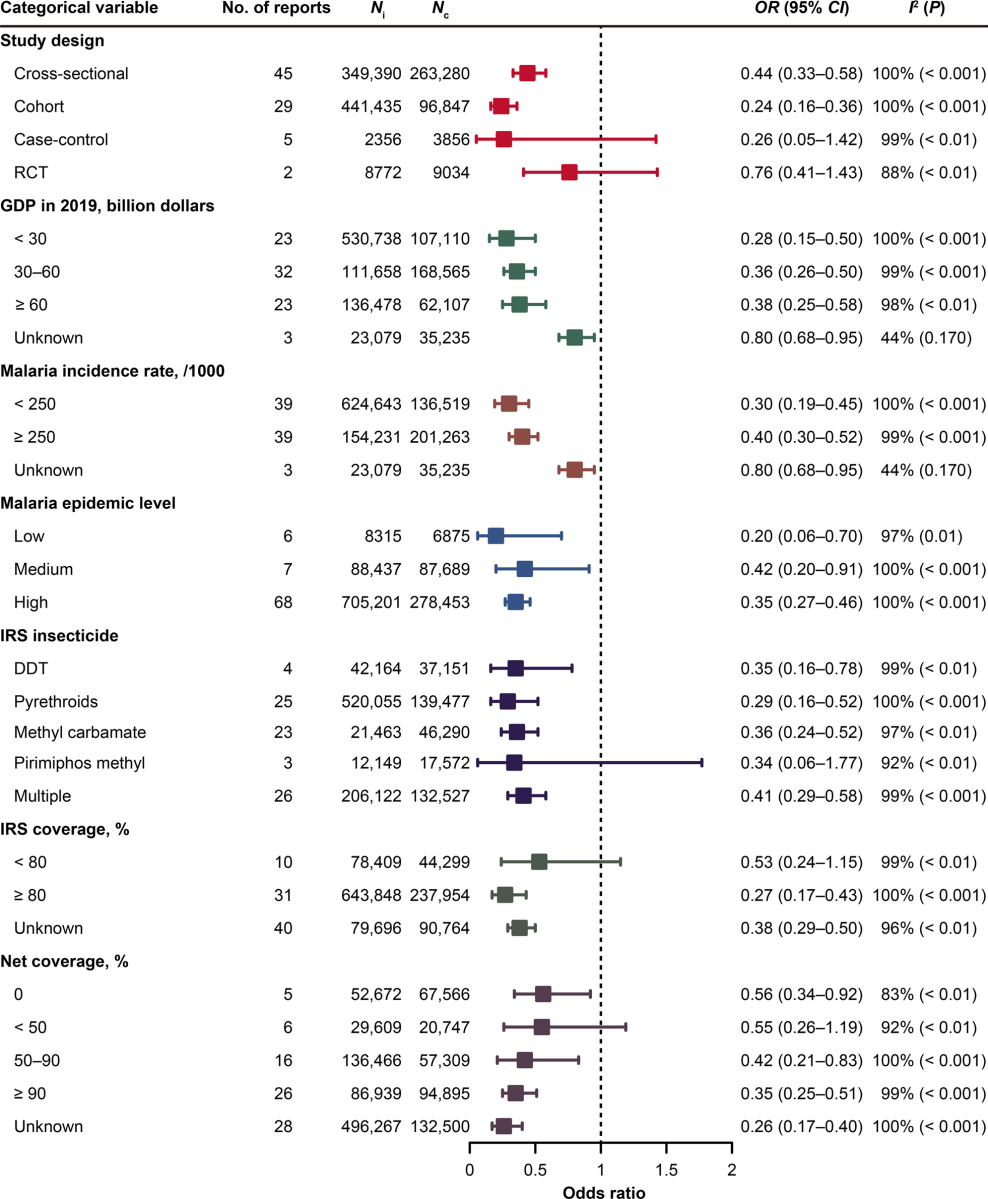
The effect of indoor residual spraying on the malaria control in subgroup analysis using the random effects model. *N*_i_: the number of people who accepted indoor residual spraying (IRS); *N*_c_: the number of people who did not accept IRS; *OR*: odds ratio; *CI*: confidence interval; *P*: *P*-value denoting the level of heterogeneity among studies; RCT: randomized controlled trial; DDT: dichloro-diphenyl-tricgloroethane

When classified by study design, 29 cohort reports and 45 cross-sectional reports showed a positive protection of IRS, with pooled *OR*s of 0.24 (95% *CI*: 0.16–0.36) and 0.44 (95% *CI*: 0.33–0.58), respectively. Five case-control reports and two RCT reports did not present statistically significant effectiveness of IRS on malaria (Fig. 4 and Additional file 1: Fig. S1).

When classified by the country’s GDP in 2019, studies in countries with a GDP < 30 billion dollars showed the best effectiveness of IRS (pooled *OR* = 0.28, 95% *CI*: 0.15–0.50), followed by that in countries with a GDP of 30–60 billion dollars (pooled *OR* = 0.36, 95% *CI*: 0.26–0.50) and a GDP ≥ 60 billion dollars (pooled *OR* = 0.38, 95% *CI*: 0.25–0.58), respectively (Fig. 4 and Additional file 1: Fig. S2).

When classified by malaria incidence rate in 2019, the highest effectiveness of IRS was observed in countries with malaria incidence rate < 250 per 1000 population at risk, while countries with rate ≥ 250 per 1000 population at risk performed slightly worse, with similar pooled *OR*s being 0.30 (95% *CI*: 0.19–0.45) and 0.40 (95% *CI*: 0.30–0.52), respectively. Similar differences also occurred in settings with different levels of malaria epidemic. Better protective effects of IRS were observed in studies reporting a low epidemic level compared to areas with a high level (pooled *OR*: 0.20 vs 0.35) in Fig. 4 and Additional file 1: Fig. S3.

When classified by IRS insecticide, except for propoxur and pirimiphos methyl (both only with 3 reports), the other insecticides involved in studies showed significant effects on the decrease of malaria incidence rate. Of these, pyrethroids had the lowest pooled *OR* of 0.29 (95% *CI*: 0.16–0.52), followed by DDT (*OR* = 0.35, 95% *CI*: 0.16–0.78) and methyl carbamate (*OR* = 0.36, 95% *CI*: 0.24–0.52) in Fig. 4 and Additional file 1: Fig. S4.

When classified by IRS coverage, it showed a stronger protective effect of IRS on the risk of malaria in the group with IRS coverage ≥ 80% with *OR* of 0.27 (95% *CI*: 0.17–0.43). In contrast, IRS coverage < 80% were not related to the decrease of malaria risk with *OR* of 0.53 (95% *CI*: 0.24–1.15). In addition, the effectiveness of IRS increased with the increase of the coverage of bed net in households. A significantly lower pooled *OR* (0.56 vs 0.35) was observed in the group of a coverage ≥ 90% (Fig. 4 and Additional file 1: Fig. S5).

### Results of meta-regression and sensitivity analysis

In the multivariate meta-regression model including all the subgroup factors, none of these factors had any significant influence on effect sizes (all *P* > 0.05) (Table 2). The results remained stable when conducting the leave-one-out sensitivity analysis (Additional file 1: Table S3). When only the 30 cross-sectional/case-control studies were kept, the overall pooled *OR* increased slightly from 0.35 (95% *CI*: 0.27–0.44) to 0.42 (95% *CI*: 0.31–0.56) (Additional file 1: Fig. S6). In the subgroup analysis within only cross-sectional/case-control studies, the most pooled estimates increased slightly. When only the eight cohort/RCT studies were kept, the pooled *RR* was 0.34 (95% *CI*: 0.23–0.49) (Additional file 1: Fig. S7). The effectiveness of IRS remained strong in most subgroup analysis.

**Table 2:** Multivariate meta-regression on the association between indoor residual spraying and malaria risk

Variable	Coefficients (95% *CI*)	*P-*value
Study design		
Case-control study	Reference	–
Cohort study	− 0.607 (− 2.344 to 1.130)	0.493
Cross-sectional study	0.323 (− 1.179 to 1.825)	0.673
RCT study	1.436 (− 1.330 to 4.202)	0.309
GDP, billion dollars		
< 30	Reference	–
30–60	0.863 (− 0.920 to 2.646)	0.343
≥ 60	0.093 (− 0.996 to 1.182)	0.867
Unknown	0.843 (− 1.925 to 3.611)	0.551
Incidence rate (/1000)		
< 250	Reference	–
≥ 250	0.286 (− 0.960 to 1.532)	0.653
IRS chemicals		
DDT	Reference	–
Pyrethroids	− 0.010 (− 1.374 to 1.354)	0.989
Methyl carbamate	0.070 (− 2.034 to 2.173)	0.948
Pirimiphos-methyl	− 0.757 (− 3.751 to 2.236)	0.620
Multiple	− 0.099 (− 1.529 to 1.332)	0.893
IRS coverage, %		
< 80	Reference	–
≥ 80	− 0.562 (− 1.699 to 0.575)	0.333
Unknown	− 0.059 (− 1.546 to 1.428)	0.938
Net coverage, %		
0	− 0.286 (− 2.461 to 1.890)	0.797
< 50	Reference	–
50–90	− 0.137 (− 1.757 to 1.482)	0.868
≥ 90	− 0.908 (− 3.282 to 1.467)	0.454
Unknown	− 0.390 (− 1.982 to 1.203)	0.632
Malaria epidemic level		
High	Reference	–
Medium	− 0.522 (− 1.890 to 0.845)	0.454
Low	− 0.793 (− 2.104 to 0.517)	0.235

*CI* confidence interval, *RCT* randomized controlled trial, *GDP* gross domestic product, *DDT* dichloro-diphenyl-tricgloroethane, *IRS* indoor residual spraying

## Discussion

In this study, we pooled the results from 38 original articles (81 reports) regarding the effectiveness of IRS implementation on the control of malaria. We identified an obvious and extensive protective effect of IRS on the control of malaria, regardless of countries’ GDP, incidence rate of malaria, IRS coverage, type of IRS insecticide, epidemic level of malaria, coverage level of bed net, and study design among the studies included in analysis. Sensitivity analyses and results of funnel plot and the Egger’s test proved that no significant publication bias existed, and our findings were reliable and robust. High heterogeneity existed in the meta-analysis of overall studies and the subgroup analyses. However, all the variables in the subgroup analysis did not show a significant correlation with the outcome indicator.

A meta-analysis published in 2012 had the same research purpose as ours, which included only 13 original papers and concluded that IRS could reduce the risk of malaria by 62% [14]. This meta-analysis also found an excessive degree of heterogeneity across original studies and indicated a high initial prevalence of malaria, multiple spraying rounds, the use of DDT, and in areas with *Plasmodium falciparum* and *P. vivax* malaria were associated with better effectiveness of the implementation of IRS. Though there were some differences in spraying year, study design, and effect size used between this meta-analysis and ours, and more than 20 extra studies have been published since 2012, our study reported a reduced risk of 65% via performing IRS, which was very close to the value in the prementioned meta-analysis. Therefore, the effectiveness of IRS has obtained further confirmation.

We found the effectiveness of IRS on malaria decreased slightly with a higher GDP of countries. It may be explained by the fact that richer countries have been providing multiple and high-quality intervention measures against malaria to their citizens for a long term. In addition, people living in a more affluent and urbanized country usually enjoy better housing conditions with other effective measures to protect them from mosquito’s bites. Therefore, countries with a high GDP might use effective alternative interventions and mask the effectiveness of IRS. Zhao et al*.* found an increased per-capita GDP might indirectly influence the reduction of malaria cases at a macro level [54], and Xu et al*.* reported a negative correlation between annual malaria incidence and national GDP [55]. Countries with a relatively low GDP should allocate locally available IRS resources properly and simultaneously apply other effective interventions to contain the malaria epidemic. In the subgroup analysis, we also found a better protective impact of IRS in countries with a lower malaria incidence rate. Due to the subtle difference existing in *OR* values across studies with different malaria incidences, we cannot conclude that IRS’s effectiveness was associated with malaria incidence.

Higher effect of IRS was found in countries and areas with IRS coverage ≥ 80%. In contrast, it was much less effective in settings with IRS coverage < 80%. This finding is consistent with some previous investigations. Elmardi et al*.* used a multilevel multivariate logistic regression model to analyze cross-sectional data, and demonstrated that a higher level of IRS coverage was associated with fewer malaria infections [56]. Another study showed a negative relationship between IRS coverage and malaria incidence but did not obtain a statistical significance [57]. It has been proved that stopping IRS in Uganda, a country with a high bed net coverage, would be faced with a fivefold increase in malaria incidence within 10 months [58]. As a result, IRS could play a critical role in achieving global malaria targets, and its coverage should be promoted as high as possible through improved community engagement [57]. Furthermore, this study upheld the WHO guidance on IRS coverage of at least 80% in order to have significant effectiveness and thereafter benefit the community.

In the subgroup analysis, DDT, pyrethroids, methyl carbamate, and combined use of multiple insecticides showed great effectiveness in controlling malaria, particularly pyrethroids. Pirimiphos-methyl did not present an obvious protective impact. Only three studies performed this IRS insecticide, therefore corresponding pooling estimates might not be accurate and reliable. This review included original reports carried out in a large time span, thus our results can only reflect the effectiveness of IRS insecticides in the past other than right now. An increased number of studies have reported the rapid spread of insecticide resistance in malaria vectors and rebounds of malaria in some endemic areas. Almost all of IRS insecticides reviewed in this study were reported to have generated or to be generating resistance among malaria vectors such as *Anopheles culicifacies*, *An. gambiae*, *An. coluzzii*, and *An. stephensi* in different countries and areas [59–64]. Therefore, the increasing resistance of IRS insecticides may pose a growing threat to malaria control, the monitoring of local insecticide resistance before implementation of IRS might be necessary to pick out an insecticide with a high sensitivity for local malaria vectors. In addition, IRS using alternative insecticide formulations may be needed.

We also observed better IRS effects in settings with a higher bed net coverage compared with settings without net. This is reasonable that comprehensive use of multiple intervention measures against malaria performs better than single use. A review published in 2009 drew a similar conclusion that combined use of IRS and nets was more protective relative to IRS alone (*OR* = 0.71 and 0.63 in two studies, respectively) [65]. Gimnig et al. found IRS could provide added benefits in an area of moderate to high transmission with moderate ITN coverage, while the value of adding ITNs to IRS remained unclear as their benefits were likely to be masked by IRS [49]. A modeling study concluded that long-lasting insecticidal net use of 56% and IRS coverage of 70% was the most cost-effective malaria control strategy in western Kenya [66]. Based on above evidence, the necessity and potential benefits of performing IRS and improving IRS coverage are further highlighted. Research on how to maximize the benefits of using two measures concurrently, particularly in the context of increasing resistance to IRS insecticides, is encouraged [49].

Some limitations should be acknowledged in this systematic review and meta-analysis. First, most of original studies were cross-sectional studies, which could only provide limited epidemiological evidence. Second, malaria definition included multiple indicators such as parasites infection, *Plasmodium falciparum* infection, malaria parasitemia, clinical malaria symptoms, and microscopic parasitemia. Inconsistent diagnostic methods and criteria might influence the comparison within these studies. Third, periods from IRS implementation to outcome measuring varied among studies, thus the effect sizes might not be comparable across them and the accuracy of pooling estimates was impacted. Fourth, the vectors and their resistance were inconsistent among countries and areas, which might lead to the underestimation of IRS’s effect. In addition, it seemed some unreasonable to observe a higher effectiveness of IRS in areas with a lower malaria incidence and epidemic level in the results, though the differences were tiny. The association between IRS effectiveness and malaria might be distorted by some confounding factors across studies such as insecticide assistance and spraying frequency. This issue is worth further investigation with confounding factors controlled.

## Conclusions

IRS showed a positive effect on the control of malaria globally. In the past decades of fighting against malaria, IRS played an essential role in killing of pathogen-carrying vectors and preventing people from infection with malaria. Effectiveness was associated with the IRS coverage and the type of IRS insecticide. Higher IRS coverage and the use of pyrethroids are key measures to reduce malaria infection, and other interventions can be supplemented in malaria prevention. However, growing insecticide resistance should be paid more attention to before the implementation of IRS. The policy makers should also consider factors concerning IRS implementation such as GDP, incidence and prevalence rate of malaria, and IRS coverage to direct the formulation of policies. More efforts should focus on increasing IRS coverage, developing more effective new insecticides against malaria and implementing multiple interventions comprehensively for specific settings in the future.

## Supplementary Information

**Additional file 1: Table S1.** Quality assessment of observational studies. **Table S2.** Quality assessment of RCT studies. **Table S3.** Sensitivity analysis by omitting each article. **Figure S1.** The effect of IRS on the malaria incidence classified by study design using the random effects model. **Figure S2.** The effect of IRS on the malaria incidence classified by GDP using the random effects model. **Figure S3.** The effect of IRS on the malaria incidence classified by malaria incidence rate (A) and malaria epidemic level (B) using the random effects model. **Figure S4.** The effect of IRS on the malaria incidence classified by IRS insecticide using the random effects model. **Figure S5.** The effect of IRS on the malaria incidence classified by IRS coverage rate (A) and bed net coverage net (B) using the random effects model. **Figure S6.** The effect of IRS on the malaria incidence in subgroup analysis using the random effects model only within cross-sectional/case-control studies. **Figure S7.** The effect of IRS on the malaria incidence in subgroup analysis using the random effects model only within cohort/RCT studies.

## Data Availability

All data generated or analyzed during this study are included in this published article and its Additional files.

## Abbreviations

IRS: Indoor residual spraying
*OR*: Odds ratio
DDT: Dichloro-diphenyl-trichloroethane
WHO: World Health Organization
PRISMA: Preferred Reporting Items for Systematic Reviews and Meta-Analyses
JBI: Joanna Briggs Institute
*RR*: Relative ratio
*IRR*: Incidence rate ratio
*RD*: Rate difference
*CI*: Confidence interval
GDP: Gross domestic product
RCT: Randomized controlled trial
