# Supplementary material for: Effectiveness of indoor residual spraying on malaria control: a systematic review and meta-analysis
Source: Infect Dis Poverty. 2022 Jul 23;11:83. doi: 10.1186/s40249-022-01005-8 (PMC9308352; doi:10.1186/s40249-022-01005-8)
Supplement: Supplementary file 1 — Additional file 1: Table S1. Quality assessment of observational studies. Table S2. Quality assessment of RCT studies. Table S3. Sensitivity analysis by omitting each article. Figure S1. The effect of IRS on the malaria incidence classified by study design using the random effects model. Figure S2. The effect of IRS on the malaria incidence classified by GDP using the random effects model. Figure S3. The effect of IRS on the malaria incidence classified by malaria incidence rate (A) and malaria epidemic level (B) using the random effects model. Figure S4. The effect of IRS on the malaria incidence classified by IRS insecticide using the random effects model. Figure S5. The effect of IRS on the malaria incidence classified by IRS coverage rate (A) and bed net coverage net (B) using the random effects model. Figure S6. The effect of IRS on the malaria incidence in subgroup analysis using the random effects model only within cross-sectional/case-control studies. Figure S7. The effect of IRS on the malaria incidence in subgroup analysis using the random effects model only within cohort/RCT studies. [file 40249_2022_1005_MOESM1_ESM.docx]

**Additional file 1**

Table S1. Quality assessment of observational studies

| **Study** | **Item 1** | **Item 2** | **Item 3** | **Item 4** | **Item 5** | **Item 6** | **Item 7** | **Item 8** | **Item 9** |
| --- | --- | --- | --- | --- | --- | --- | --- | --- | --- |
| Jambou et al. | Yes | Yes | Yes | Yes | Unclear | No | Yes | Yes | Include |
| Guyatt et al. | Yes | Yes | Yes | Yes | Unclear | No | Yes | Yes | Include |
| Gunasekaran et al. | Yes | Yes | Yes | Yes | Yes | Yes | Yes | Yes | Include |
| Bukirwa et al. | Yes | Yes | Yes | Yes | Yes | Yes | Yes | Yes | Include |
| Aregawi et al. | Yes | Yes | Unclear | Yes | Yes | Yes | Yes | Yes | Include |
| Hamusse et al. | Yes | Yes | Yes | Yes | Unclear | No | Unclear | Yes | Include |
| Fullman et al. | Yes | Yes | Yes | Unclear | Yes | Yes | Yes | Yes | Include |
| Gimnig et al. | Yes | Yes | Yes | Unclear | Yes | Yes | Yes | Yes | Include |
| Hamainza et al. | Yes | Yes | Yes | Yes | Unclear | Unclear | Yes | Yes | Include |
| Hast et al. | Yes | Yes | Yes | Yes | Unclear | No | Yes | Yes | Include |
| Arinaitwe et al. | Yes | Yes | No | Unclear | Yes | Yes | Unclear | Yes | Include |
| Habyarimana et al. | No | Yes | Unclear | Yes | Yes | Yes | Unclear | Yes | Include |
| Kamya et al. | Yes | Yes | Yes | No | Yes | Yes | Yes | Yes | Include |
| Fekadu et al. | Yes | Yes | No | Unclear | Yes | Yes | Yes | Yes | Include |
| Mashauri et al. | Yes | Yes | Yes | Yes | No | No | Yes | Yes | Include |
| West et al. | Yes | Yes | Yes | Yes | Yes | Yes | Yes | Yes | Include |
| Kesteman et al. | Yes | Yes | Yes | Yes | Yes | Unclear | Yes | Yes | Include |
| Skarbinski et al. | Yes | Yes | Yes | Yes | Yes | Yes | Yes | Yes | Include |
| Sintasath et al. | Yes | Yes | No | Yes | NA | NA | Yes | Yes | Include |
| Wubishet et al. | Yes | Yes | Yes | Yes | Yes | Yes | Yes | Yes | Include |
| Kleinschmidt et al. | Yes | Yes | Unclear | Yes | Yes | Yes | Yes | Yes | Include |
| Tseng et al. | Yes | Yes | Yes | No | No | No | Yes | Unclear | Include |
| Protopopoff et al. | Yes | Yes | Yes | Yes | Yes | Yes | Yes | Yes | Include |
| Rek et al. | Yes | Yes | Unclear | Yes | Yes | Yes | Yes | Unclear | Include |
| Steinhardt et al. | Yes | Yes | Yes | Yes | Yes | Yes | Yes | Yes | Include |
| Kesteman et al. | Yes | Yes | Unclear | Yes | Yes | Yes | Yes | Yes | Include |
| Odugbemi et al. | Yes | Yes | Unclear | Yes | Yes | Yes | Yes | Yes | Include |
| Raouf et al. | Yes | Unclear | Unclear | Yes | No | No | Yes | Yes | Include |
| Nankabirwa et al. | Yes | Yes | Unclear | Yes | Yes | Yes | Yes | Yes | Include |
| Neeru et al. | Yes | Yes | Yes | Yes | Yes | Yes | Yes | Yes | Include |
| Rehman et al. | Yes | Yes | Yes | Yes | Yes | Yes | Yes | Yes | Include |
| Zhou et al. | Yes | No | Yes | Yes | No | No | Yes | Yes | Include |
| Wanzira et al. | Yes | Yes | Yes | Yes | Yes | Yes | Yes | Yes | Include |
| Smith et al. | Yes | Yes | Yes | Yes | Yes | Yes | Yes | Yes | Include |
| Siegert et al. | Yes | Yes | Yes | Yes | Yes | Yes | Yes | Yes | Include |
| Tugume et al. | Yes | Yes | Yes | Yes | No | No | Yes | Yes | Include |

Note:

Item 1: Were the criteria for inclusion in the sample clearly defined?

Item 2: Were the study subjects and the setting described in detail?

Item 3: Was the exposure measured in a valid and reliable way?

Item 4: Were objective, standard criteria used for measurement of the condition?

Item 5: Were confounding factors identified?

Item 6: Were strategies to deal with confounding factors stated?

Item 7: Were the outcomes measured in a valid and reliable way?

Item 8: Was appropriate statistical analysis used?

Item 9: Overall appraisal

Table S2. Quality assessment of RCT studies

| **Study** | **Item 1** | **Item 2** | **Item 3** | **Item 4** | **Item 5** | **Item 6** | **Item 7** | **Item 8** | **Item 9** | **Item 10** |
| --- | --- | --- | --- | --- | --- | --- | --- | --- | --- | --- |
| Chaccour et al. | Yes | Yes | Yes | Yes | Unclear | Yes | Yes | Yes | Yes | Include |
| Loha et al. | Yes | Yes | Yes | Yes | Yes | Yes | Yes | Yes | Yes | Include |

Note:

Item 1: Is it clear in the study what is the cause and what is the effect (i.e. there is no confusion about which variable comes first)?

Item 2: Were the participants included in any comparisons similar?

Item 3: Were the participants included in any comparisons receiving similar treatment/care, other than the exposure or intervention of interest?

Item 4: Was there a control group?

Item 5: Were there multiple measurements of the outcome both pre and post the intervention/exposure?

Item 6: Was follow up complete and if not, were differences between groups in terms of their follow up adequately described and analyzed?

Item 7: Were the outcomes of participants included in any comparisons measured in the same way?

Item 8: Were outcomes measured in a reliable way?

Item 9: Was appropriate statistical analysis used?

Item 10: Overall appraisal

Table S3. Sensitivity analysis by omitting each article

| **Study** | ***OR*** | **95% *CI*** | ***P*** | **tau^2^** | ***I*^2^** |
| --- | --- | --- | --- | --- | --- |
| Omitting Jambou et al. (2001) | 0.3496 | (0.2723–0.4487) | < 0.0001 | 1.2529 | 99.70% |
| Omitting Guyatt et al. (2002) | 0.3477 | (0.2707–0.4465) | < 0.0001 | 1.2592 | 99.70% |
| Omitting Gunasekaran et al. (2005) | 0.3484 | (0.2713–0.4474) | < 0.0001 | 1.2579 | 99.70% |
| Omitting Sintasath et al. (2005) | 0.3476 | (0.2706–0.4464) | < 0 .0001 | 1.2593 | 99.70% |
| Omitting Neeru Singh et al. (2006) | 0.3467 | (0.2699–0.4454) | < 0.0001 | 1.2619 | 99.70% |
| Omitting Kleinschmidt et al. (2006 | 0.3441 | (0.2678–0.4419) | < 0.0001 | 1.2600 | 99.70% |
| Omitting Protopopoff et al. (2008) | 0.3439 | (0.2677–0.4417) | < 0.0001 | 1.2596 | 99.70% |
| Omitting Protopopoffet al. (2008) | 0.3435 | (0.2675–0.4412) | < 0.0001 | 1.2585 | 99.70% |
| Omitting Tseng et al. (2008) | 0.3479 | (0.2709–0.4469) | < 0.0001 | 1.2595 | 99.70% |
| Omitting Tseng et al. (2008) | 0.3502 | (0.2729–0.4494) | < 0.0001 | 1.2499 | 99.70% |
| Omitting Tseng et al. (2008) | 0.3510 | (0.2736–0.4502) | < 0.0001 | 1.2451 | 99.70% |
| Omitting Tseng et al. (2008) | 0.3543 | (0.2770–0.4532) | < 0.0001 | 1.2162 | 99.60% |
| Omitting Tseng et al. (2008) | 0.3577 | (0.2808–0.4556) | < 0.0001 | 1.1738 | 99.60% |
| Omitting Tseng et al. (2008) | 0.3584 | (0.2817–0.4560) | < 0.0001 | 1.1626 | 99.60% |
| Omitting Bukirwa et al. (2009) | 0.3467 | (0.2699–0.4454) | < 0.0001 | 1.2619 | 99.70% |
| Omitting Bukirwa et al. (2009) | 0.3464 | (0.2696–0.4450) | < 0.0001 | 1.2622 | 99.70% |
| Omitting Bukirwa et al. (2009) | 0.3410 | (0.2658–0.4374) | < 0.0001 | 1.2453 | 99.70% |
| Omitting Bukirwa et al. (2009) | 0.3401 | (0.2653–0.4360) | < 0.0001 | 1.2384 | 99.60% |
| Omitting Zhou et al. (2010) | 0.3365 | (0.2636–0.4295) | < 0.0001 | 1.1959 | 99.70% |
| Omitting Rehmanet al. (2011) | 0.3404 | (0.2655–0.4364) | < 0.0001 | 1.2407 | 99.70% |
| Omitting Rehmanet al. (2011) | 0.3442 | (0.2680–0.4422) | < 0.0001 | 1.2605 | 99.70% |
| Omitting Aregawi et al. (2011) | 0.3646 | (0.2899–0.4586) | < 0.0001 | 1.0495 | 99.60% |
| Omitting Hamusse et al. (2011) | 0.3430 | (0.2671–0.4405) | < 0.0001 | 1.2567 | 99.70% |
| Omitting Skarbinski et al. (2012) | 0.3510 | (0.2737–0.4502) | < 0.0001 | 1.2441 | 99.70% |
| Omitting Fullman et al. (2013) | 0.3418 | (0.2664–0.4386) | < 0.0001 | 1.2500 | 99.70% |
| Omitting Fullman et al. (2013) | 0.3422 | (0.2666–0.4393) | < 0.0001 | 1.2529 | 99.70% |
| Omitting Fullman et al. (2013) | 0.3460 | (0.2694–0.4443) | < 0.0001 | 1.2596 | 99.70% |
| Omitting Steinhardt et al. (2013) | 0.3456 | (0.2690–0.4440) | < 0.0001 | 1.2622 | 99.70% |
| Omitting Mashauri et al. (2013) | 0.3442 | (0.2680–0.4421) | < 0.0001 | 1.2600 | 99.70% |
| Omitting Mashauri et al. (2013) | 0.3461 | (0.2694–0.4446) | < 0.0001 | 1.2620 | 99.70% |
| Omitting Mashauri et al. (2013) | 0.3436 | (0.2676–0.4413) | < 0.0001 | 1.2586 | 99.70% |
| Omitting Mashauri et al. (2013) | 0.3496 | (0.2724–0.4488) | < 0.0001 | 1.2525 | 99.70% |
| Omitting Mashauri et al. (2013) | 0.3440 | (0.2678–0.4419) | < 0.0001 | 1.2598 | 99.70% |
| Omitting Mashauri et al. (2013) | 0.3403 | (0.2654–0.4362) | < 0.0001 | 1.2395 | 99.70% |
| Omitting Mashauri et al. (2013) | 0.3448 | (0.2684–0.4429) | < 0.0001 | 1.2610 | 99.70% |
| Omitting Mashauri et al. (2013) | 0.3413 | (0.2660–0.4378) | < 0.0001 | 1.2472 | 99.70% |
| Omitting Mashauri et al. (2013) | 0.3448 | (0.2684–0.4430) | < 0.0001 | 1.2614 | 99.70% |
| Omitting West et al. (2013) | 0.3457 | (0.2691–0.4441) | < 0.0001 | 1.2623 | 99.70% |
| Omitting Gimnig et al. (2016) | 0.3443 | (0.2681–0.4421) | < 0.0001 | 1.2591 | 99.70% |
| Omitting Gimnig et al. (2016) | 0.3460 | (0.2693–0.4445) | < 0.0001 | 1.2622 | 99.70% |
| Omitting Gimnig et al. (2016) | 0.3458 | (0.2692–0.4443) | < 0.0001 | 1.2618 | 99.70% |
| Omitting Hamainza et al. (2016) | 0.3419 | (0.2664–0.4387) | < 0.0001 | 1.2508 | 99.70% |
| Omitting Kesteman et al. (2016) | 0.3382 | (0.2644–0.4327) | < 0.0001 | 1.2188 | 99.70% |
| Omitting Odugbemi et al. (2016) | 0.3477 | (0.2709–0.4462) | < 0.0001 | 1.2560 | 99.70% |
| Omitting Kesteman et al. (2016) | 0.3440 | (0.2679–0.4417) | < 0.0001 | 1.2582 | 99.70% |
| Omitting Kesteman et al. (2016) | 0.3405 | (0.2656–0.4366) | < 0.0001 | 1.2409 | 99.70% |
| Omitting Wanzira et al. (2017) | 0.3367 | (0.2637–0.4299) | < 0.0001 | 1.1994 | 99.70% |
| Omitting Raouf et al. (2017) | 0.3454 | (0.2689–0.4438) | < 0.0001 | 1.2622 | 99.70% |
| Omitting Raouf et al. (2017) | 0.3463 | (0.2695–0.4449) | < 0.0001 | 1.2622 | 99.70% |
| Omitting Raouf et al. (2017) | 0.3403 | (0.2654–0.4362) | < 0.0001 | 1.2397 | 99.60% |
| Omitting Rek et al. (2018) | 0.3459 | (0.2693–0.4445) | < 0.0001 | 1.2623 | 99.70% |
| Omitting Hast et al. (2019) | 0.3437 | (0.2676–0.4414) | < 0.0001 | 1.2588 | 99.70% |
| Omitting Nankabirwa et al. (2019) | 0.3444 | (0.2682–0.4424) | < 0.0001 | 1.2605 | 99.70% |
| Omitting Nankabirwa et al. (2019) | 0.3425 | (0.2668–0.4397) | < 0.0001 | 1.2543 | 99.70% |
| Omitting Nankabirwa et al. (2019) | 0.3396 | (0.2651–0.4350) | < 0.0001 | 1.2325 | 99.70% |
| Omitting Nankabirwa et al. (2019) | 0.3456 | (0.2690–0.4440) | < 0.0001 | 1.2618 | 99.70% |
| Omitting Nankabirwa et al. (2019) | 0.3448 | (0.2684–0.4430) | < 0.0001 | 1.2614 | 99.70% |
| Omitting Nankabirwa et al. (2019) | 0.3410 | (0.2659–0.4372) | < 0.0001 | 1.2436 | 99.70% |
| Omitting Nankabirwa et al. (2019) | 0.3490 | (0.2718–0.4480) | < 0.0001 | 1.2550 | 99.70% |
| Omitting Nankabirwa et al. (2019) | 0.3486 | (0.2715–0.4476) | < 0.0001 | 1.2572 | 99.70% |
| Omitting Nankabirwa et al. (2019) | 0.3461 | (0.2696–0.4444) | < 0.0001 | 1.2586 | 99.70% |
| Omitting Nankabirwa et al. (2019) | 0.3472 | (0.2703–0.4460) | < 0.0001 | 1.2609 | 99.70% |
| Omitting Nankabirwa et al. (2019) | 0.3454 | (0.2689–0.4438) | < 0.0001 | 1.2621 | 99.70% |
| Omitting Nankabirwa et al. (2019) | 0.3439 | (0.2677–0.4416) | < 0.0001 | 1.2591 | 99.70% |
| Omitting Nankabirwa et al. (2019) | 0.3490 | (0.2718–0.4481) | < 0.0001 | 1.2554 | 99.70% |
| Omitting Nankabirwa et al. (2019) | 0.3477 | (0.2707–0.4466) | < 0.0001 | 1.2601 | 99.70% |
| Omitting Nankabirwa et al. (2019) | 0.3466 | (0.2698–0.4452) | < 0.0001 | 1.2615 | 99.70% |
| Omitting Nankabirwa et al. (2019) | 0.3519 | (0.2746–0.4510) | < 0.0001 | 1.2378 | 99.70% |
| Omitting Nankabirwa et al. (2019) | 0.3510 | (0.2737–0.4502) | < 0.0001 | 1.2448 | 99.70% |
| Omitting Nankabirwa et al. (2019) | 0.3491 | (0.2719–0.4482) | < 0.0001 | 1.2546 | 99.70% |
| Omitting Loha et al. (2019) | 0.3412 | (0.2660–0.4377) | < 0.0001 | 1.2464 | 99.70% |
| Omitting Tugume et.al (2019) | 0.3413 | (0.2660–0.4379) | < 0.0001 | 1.2475 | 99.70% |
| Omitting Arinaitwe et al. (2020) | 0.3522 | (0.2751–0.4510) | < 0.0001 | 1.2312 | 99.70% |
| Omitting Habyarimana et al. (2020) | 0.3401 | (0.2653–0.4359) | < 0.0001 | 1.2380 | 99.70% |
| Omitting Kamya et al. (2020) | 0.3501 | (0.2729–0.4491) | < 0.0001 | 1.2487 | 99.70% |
| Omitting Kamya et al. (2020) | 0.3549 | (0.2779–0.4532) | < 0.0001 | 1.2052 | 99.70% |
| Omitting Wubishet et al. (2021) | 0.3439 | (0.2678–0.4417) | < 0.0001 | 1.2588 | 99.70% |
| Omitting Smith et al. (2021) | 0.3425 | (0.2668–0.4398) | < 0.0001 | 1.2543 | 99.70% |
| Omitting Siegert et al. (2021) | 0.3584 | (0.2817–0.4559) | < 0.0001 | 1.1632 | 99.70% |
| Omitting Chaccour et al. (2021) | 0.3440 | (0.2679–0.4419) | < 0.0001 | 1.2594 | 99.70% |
| Omitting Fekadu et al. (2021) | 0.3537 | (0.2764–0.4526) | < 0.0001 | 1.2216 | 99.70% |

Note:

Abbreviations: *OR*, odds ratio; *CI*, confidence interval; *P*, p-value.


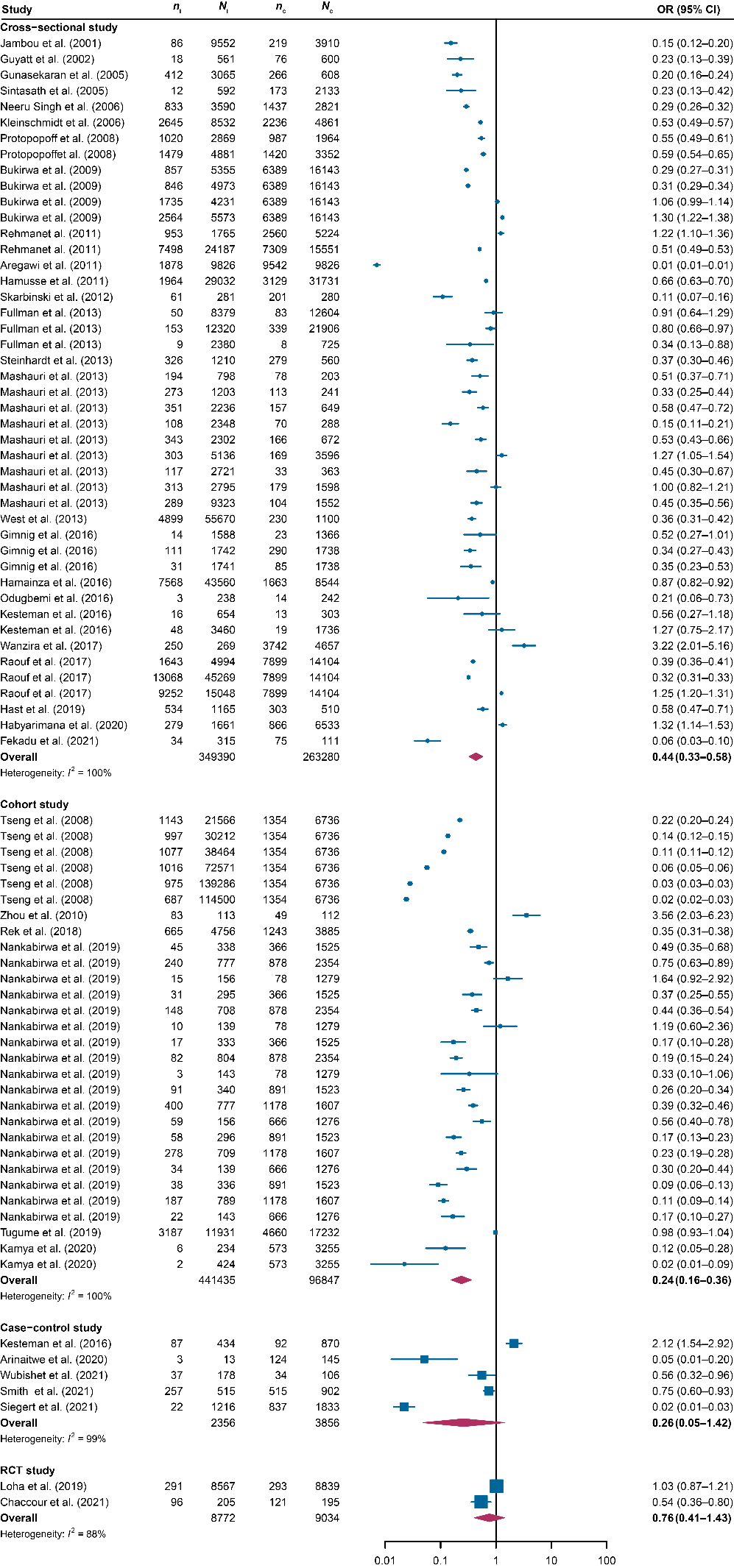


Figure S1. The effect of IRS on the malaria incidence classified by study design using the random effects model

Note:

Abbreviations: IRS, indoor residual spraying; n_i_, the number of malaria cases who accepted IRS; N_i_, the number of people who accepted IRS; n_c_, the number of malaria cases who did not accepted IRS; N_c_, the number of people who did not accept IRS; *OR*, odds ratio; *CI*, confidence interval.


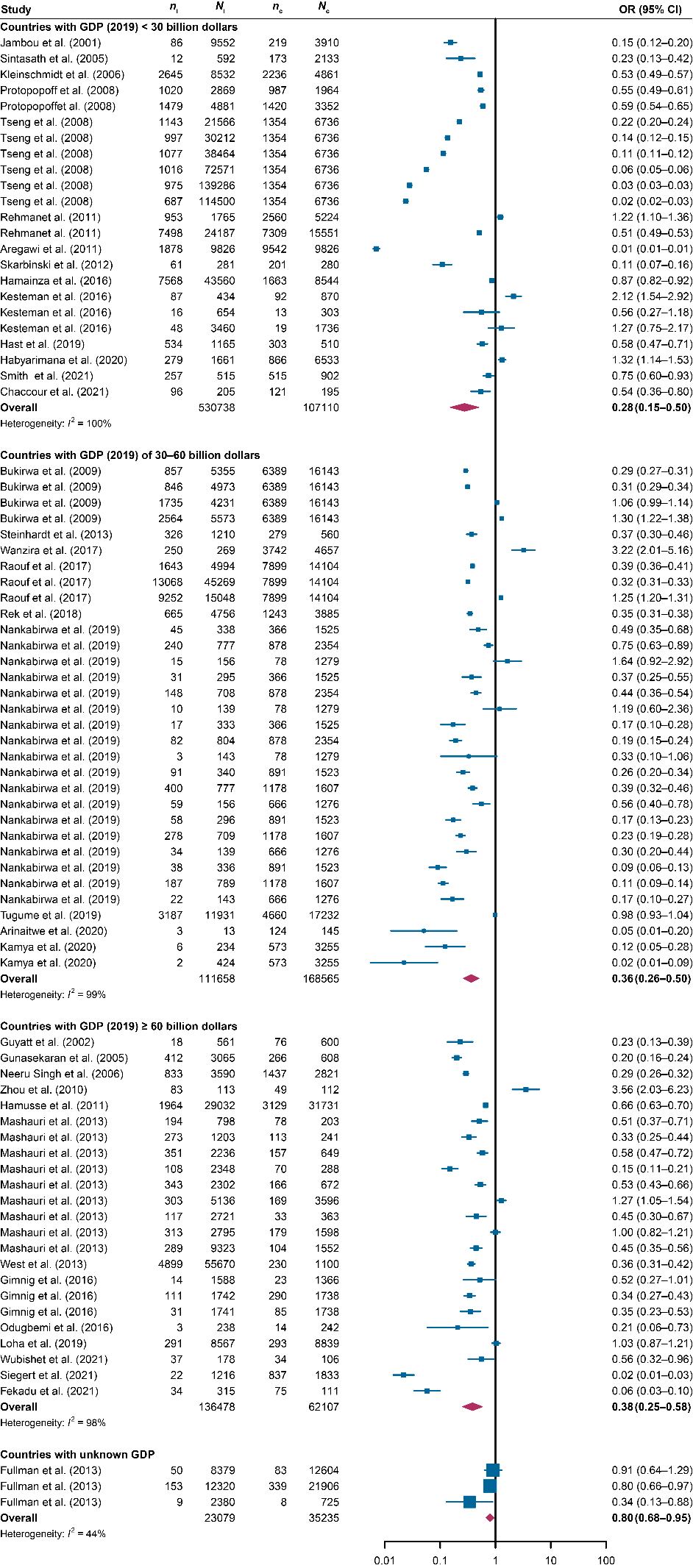


Figure S2. The effect of IRS on the malaria incidence classified by GDP using the random effects model

Note:

Abbreviations: IRS, indoor residual spraying; GDP, gross domestic product; n_i_, the number of malaria cases who accepted IRS; N_i_, the number of people who accepted IRS; n_c_, the number of malaria cases who did not accepted IRS; N_c_, the number of people who did not accept IRS; *OR*, odds ratio; *CI*, confidence interval.


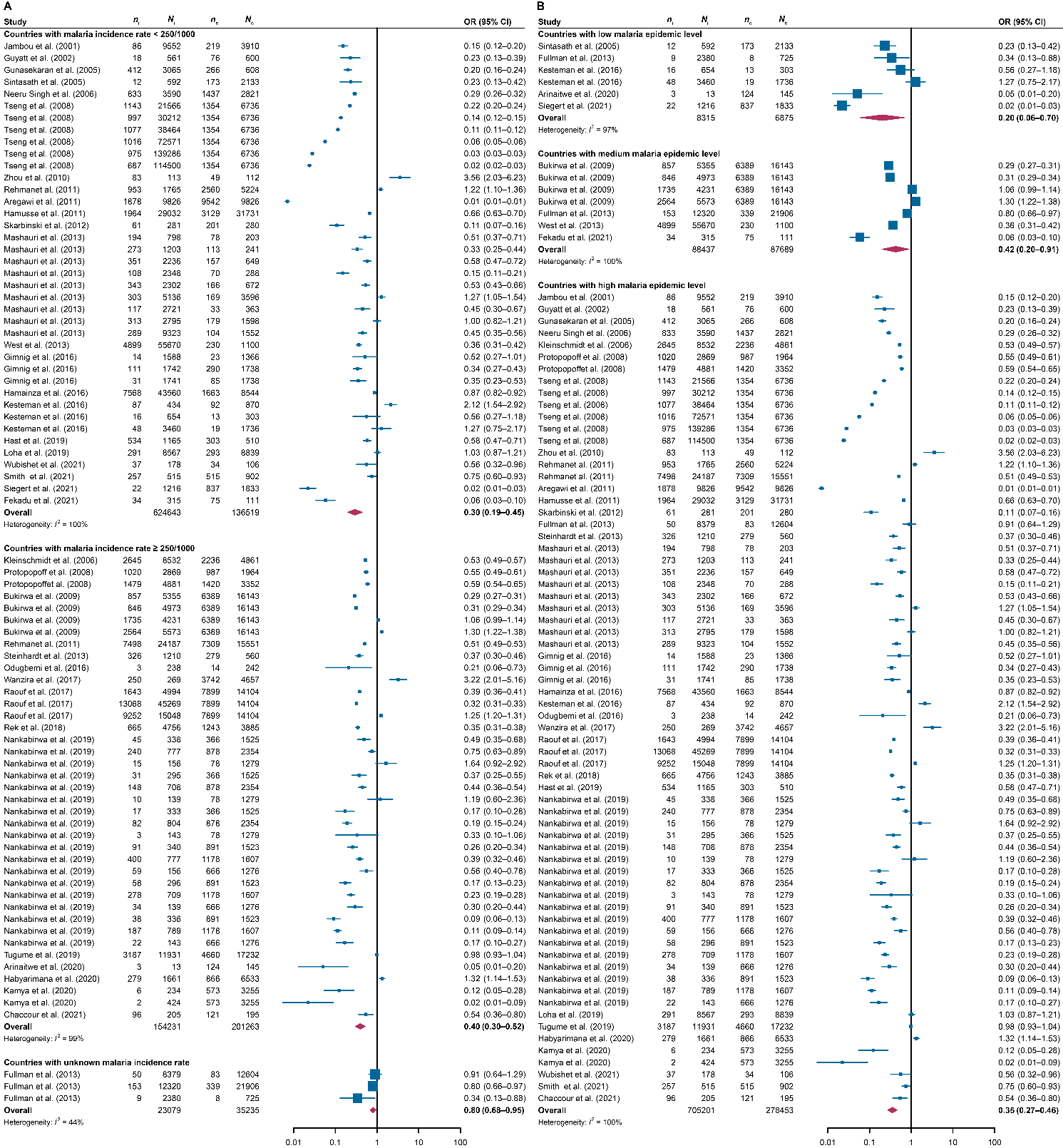


Figure S3. The effect of IRS on the malaria incidence classified by malaria incidence rate (A) and malaria epidemic level (B) using the random effects model

Note:

Abbreviations: IRS, indoor residual spraying; n_i_, the number of malaria cases who accepted IRS; N_i_, the number of people who accepted IRS; n_c_, the number of malaria cases who did not accepted IRS; N_c_, the number of people who did not accept IRS; *OR*, odds ratio; *CI*, confidence interval.


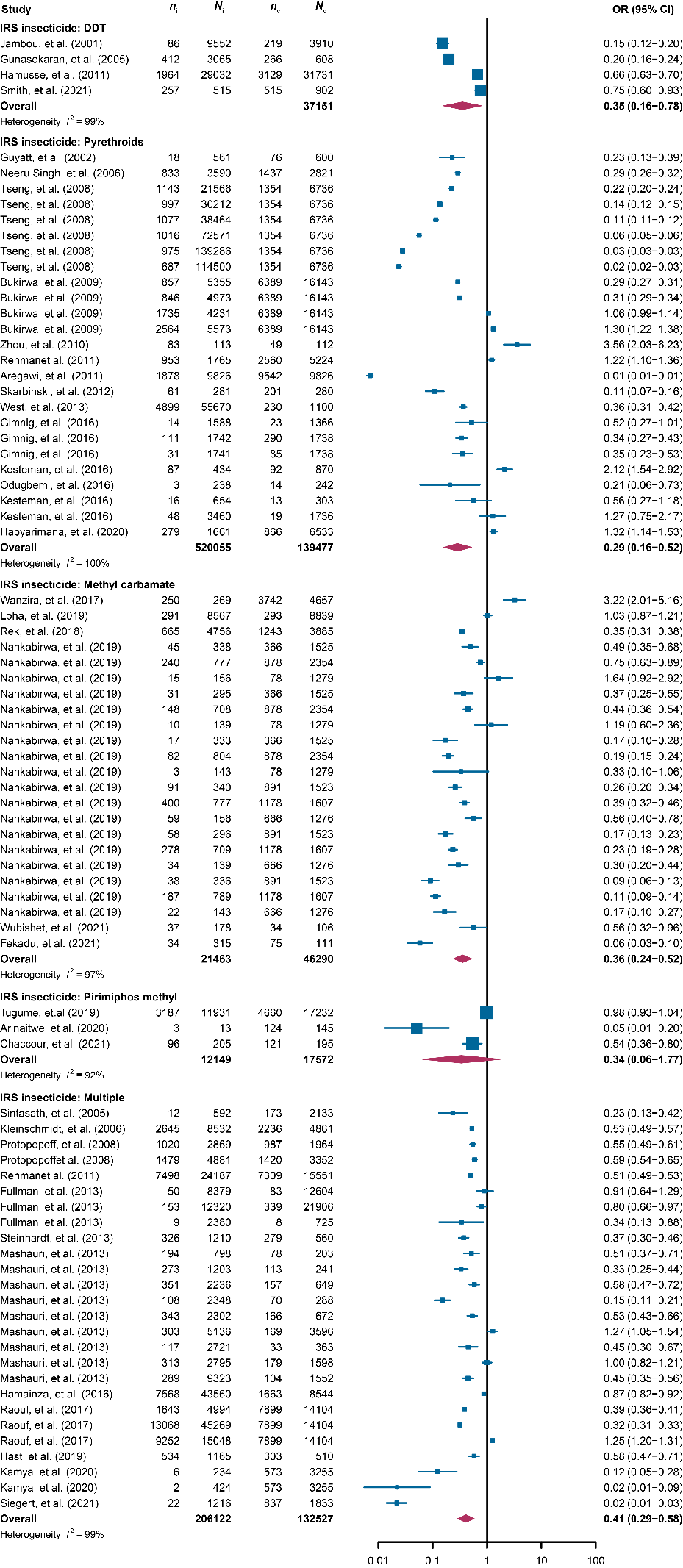


Figure S4. The effect of IRS on the malaria incidence classified by IRS insecticide using the random effects model

Note:

Abbreviations: IRS, indoor residual spraying; n_i_, the number of malaria cases who accepted IRS; N_i_, the number of people who accepted IRS; n_c_, the number of malaria cases who did not accepted IRS; N_c_, the number of people who did not accept IRS; *OR*, odds ratio; *CI*, confidence interval; DDT, Dichloro-Diphenyl-Tricgloroethane.


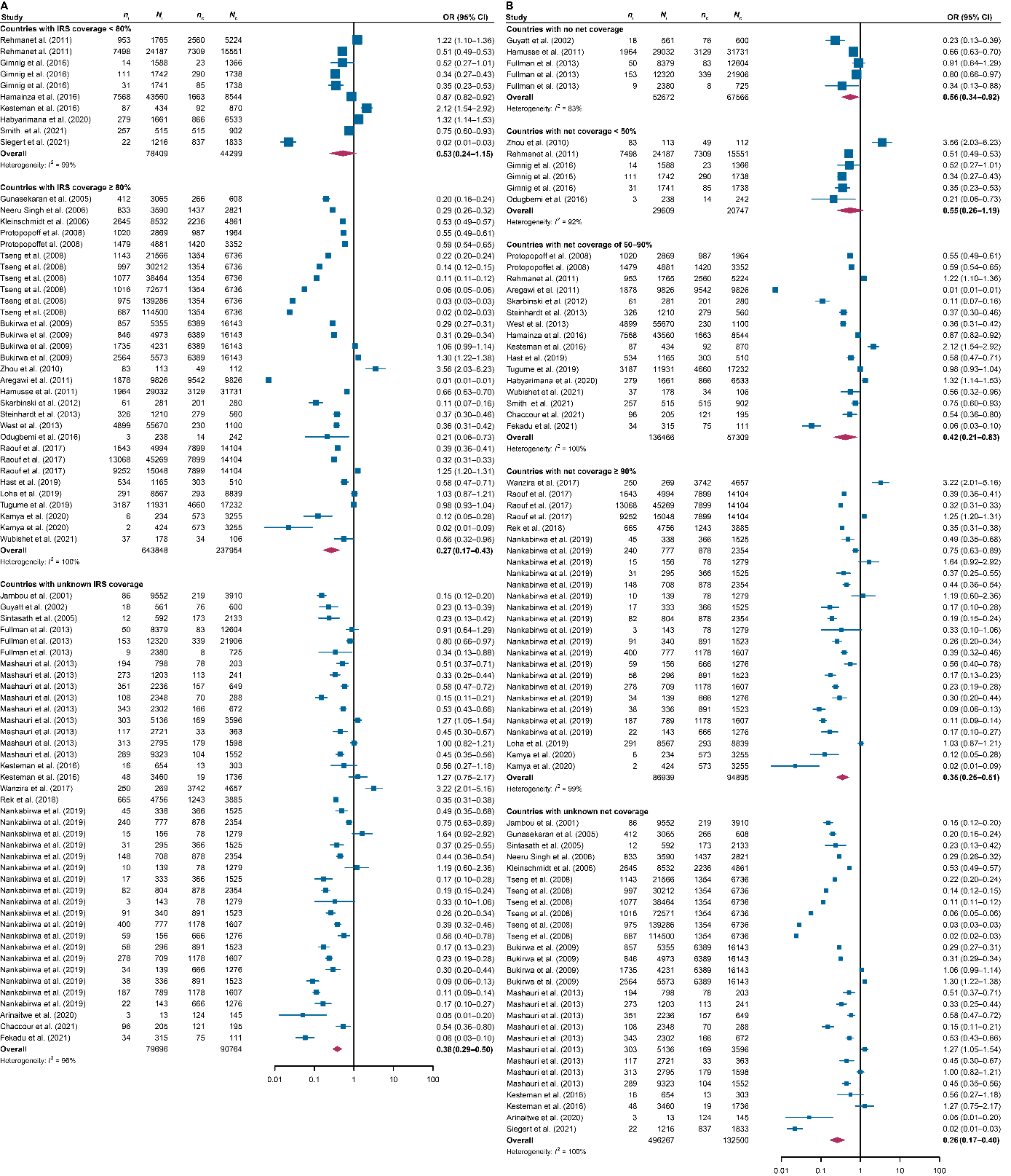


Figure S5. The effect of IRS on the malaria incidence classified by IRS coverage rate (A) and bed net coverage net (B) using the random effects model

Note:

Abbreviations: IRS, indoor residual spraying; n_i_, the number of malaria cases who accepted IRS; N_i_, the number of people who accepted IRS; n_c_, the number of malaria cases who did not accepted IRS; N_c_, the number of people who did not accept IRS; *OR*, odds ratio; *CI*, confidence interval.


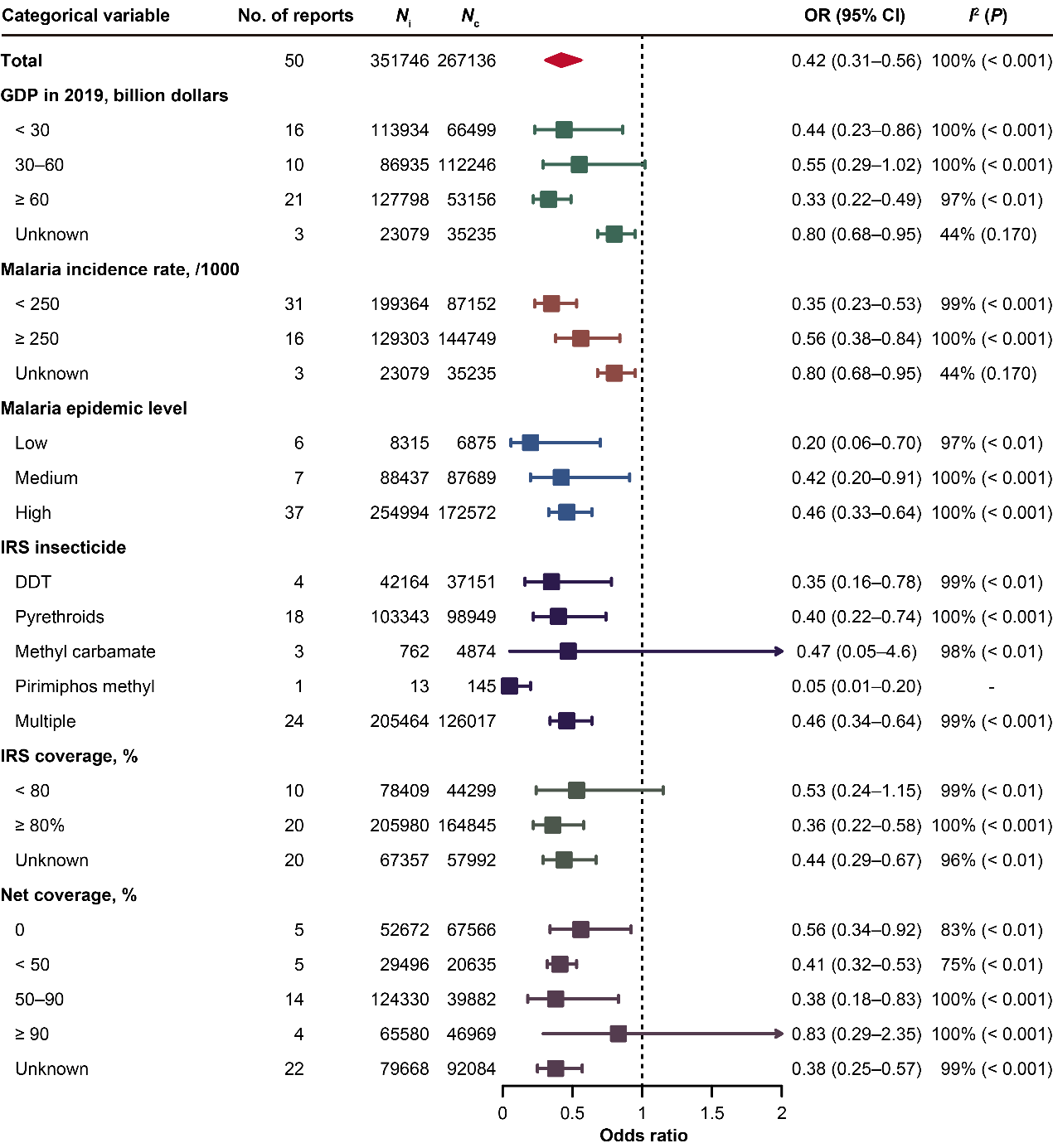


Figure S6. The effect of IRS on the malaria incidence in subgroup analysis using the random effects model only within cross-sectional/case-control studies

Abbreviations: IRS, indoor residual spraying; *N*_i_, the number of people who accepted IRS; *N*_c_, the number of people who did not accept IRS; *OR*, odds ratio; *CI*, confidence interval; *P*, p-value denoting the level of heterogeneity among studies; GDP, gross domestic product; DDT, Dichloro-Diphenyl-Tricgloroethane.


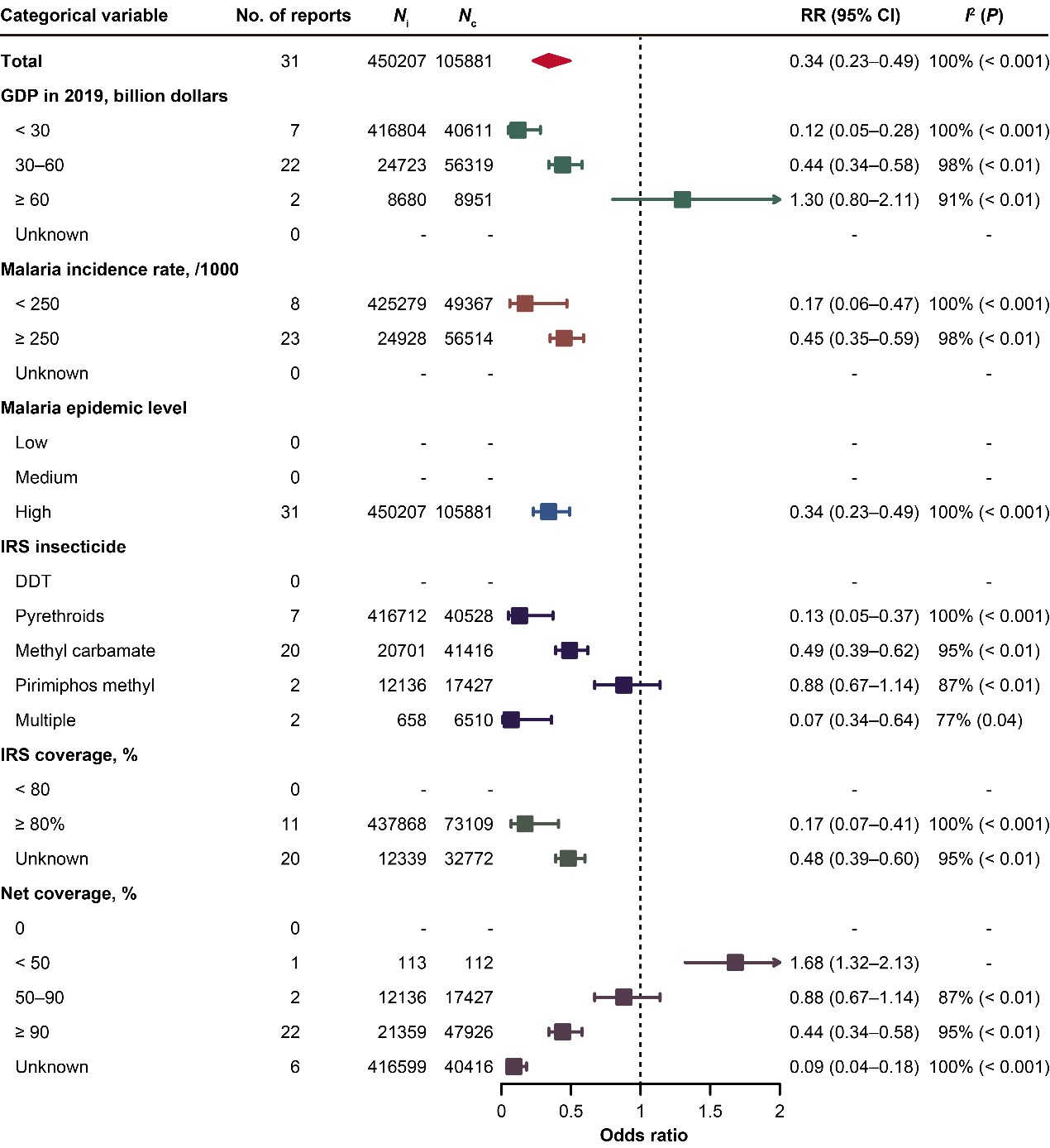


Figure S7. The effect of IRS on the malaria incidence in subgroup analysis using the random effects model only within cohort/RCT studies

Abbreviations: IRS, indoor residual spraying; n_i_, the number of malaria cases who accepted IRS; N_i_, the number of people who accepted IRS; n_c_, the number of malaria cases who did not accept IRS; N_c_, the number of people who did not accept IRS; *RR*, risk ratio; *CI*, confidence interval; *P*, p-value denoting the level of heterogeneity among studies; GDP, gross domestic product; RCT, randomized controlled trial.
